# The Molecular and Immunological Landscape of Meningiomas

**DOI:** 10.3390/ijms25179631

**Published:** 2024-09-05

**Authors:** Catharina Lotsch, Rolf Warta, Christel Herold-Mende

**Affiliations:** Division of Experimental Neurosurgery, Department of Neurosurgery, University Hospital Heidelberg, 69120 Heidelberg, Germany; catharina.lotsch@med.uni-heidelberg.de (C.L.); rolf.warta@med.uni-heidelberg.de (R.W.)

**Keywords:** meningioma, molecular profiling, DNA methylation, immunobiology, infiltrating immune cells, immunotherapy, meningioma prognosis, brain tumor diagnostics

## Abstract

Meningiomas are the most common primary intracranial tumors in adults and typically have a slow-growing and benign nature. However, there is also a substantial subset of meningiomas that shows aggressive clinical behavior and is refractory to standard treatment modalities, which are still limited to surgery and/or radiotherapy. Despite intensive research, no systemic treatment options are yet available in the clinic for these challenging tumors, resulting in poor patient outcome. Intensive research on the molecular pathogenesis of meningiomas has led to improved diagnostic tools, but so far there is no standardized implementation for the molecular profiling of these tumors for clinical practice. Recent research advances have also focused on the immunophenotyping of meningiomas, leading to several clinical trials examining the use of immune checkpoint blockade therapy in patients with clinically aggressive subtypes. In this review, we aim to summarize the current knowledge on the molecular and immunological landscape of meningiomas in detail and provide current and progressive ideas for future directions.

## 1. Introduction

Meningiomas (MGMs) represent the most common primary brain malignancies in adults, accounting for more than one-third of all central nervous system (CNS) neoplasms with a reported incidence of 8.03/100,000 in the United States [1,2]. These tumors are more commonly discovered in the elderly (>70 years of age) and occur more frequently in women with a two-fold to three-fold higher frequency than in men [3]. Meningiomas originate most likely from arachnoid cells in the meninges, which surround the brain and the spinal cord and consist of the pia, arachnoid and dura mater [4]. Fortunately, the majority of meningiomas are of a slow-growing and benign nature, but there is also a substantial subset of tumors exhibiting aggressive clinical behavior. Standard-of-care treatment modalities are still limited to surgery and (adjuvant) radiation therapy, and despite tremendous research efforts, no FDA-approved therapies are yet available in the clinic for the systemic treatment of aggressive meningiomas [5]. With increasing insights into the molecular drivers of meningioma pathogenesis and the immunobiology of the tumor, targeted therapies as well as immunotherapies are now largely the focus of research and clinical trials [6]. In this review, we aim to provide an overview on the current state of research regarding the molecular profile and the immunological landscape of meningiomas and emphasize recent and advanced investigations that could influence the clinical management of these tumors in the future.

## 2. Clinical Presentation

**Figure 1:**
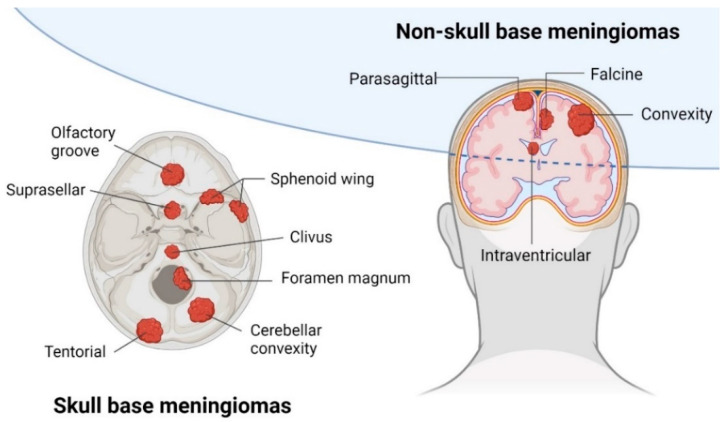
Anatomical locations of meningiomas. Schematic overview of common anatomical locations of meningiomas around the skull, with a distinction between skull-base and non-skull-base meningiomas [9].

Meningiomas occur in different anatomical locations around the skull and the spinal cord (see Figure 1). The anatomical location presents an important prognostic factor for meningioma patients as it partially determines the extent of resection during surgery [7]. The extent of surgical resection is classified in Simpson Grades I–V, with gross total resection, including Simpson Grade I–III, and subtotal resection, including Simpson Grade IV–V. Due to restricted surgical access and close proximity to critical neurovascular structures, skull base and intraventricular meningiomas are usually less amenable to gross total resection when compared to meningiomas located in the convexity [7]. In these difficult cases, radiation therapy is an essential part of the clinical management of meningioma patients, either through stereotactic radiosurgery or external beam radiotherapy. Moreover, for meningiomas that harbor a high risk of recurrence, radiation therapy is widely applied in the adjuvant setting [8].

The classification of meningiomas by the World Health Organization (WHO) is traditionally based on histopathology, with a primary focus on histopathological and cytomorphological features such as histological appearance, proliferation index and brain invasion. Meningiomas are categorized into three WHO grades (WHO°) and 15 histological subtypes (see Table 1) [4]. The relative prevalences of meningiomas have been reported to be 70–80% for WHO°1, 15–20% for atypical WHO°2 and 1–3% for anaplastic WHO°3 tumors [3,10]. Both histopathological features and the extent of resection present important factors for the prognosis of meningioma patients who have undergone surgery. In large-scale retrospective studies, recurrence rates for meningiomas have been reported to be 10–15% at 5 years for WHO°1 MGMs and up to 50% at 5 years for WHO°2 MGMs. Patients with WHO°3 MGMs face the worst prognosis with an almost inevitable risk of tumor recurrence of 90% at 5 years despite aggressive treatment efforts [7,10]. The median recurrence-free survival of patients has been reported to be 12.5 years for WHO°1 MGMs, 6.9 years for WHO°2 MGMs and only 2.4 years for WHO°3 MGMs [7]. For meningiomas, the diagnosed WHO grade is usually highly associated with its tumor-specific mortality, which means that patients with a WHO°1 MGM are expected to have a lower risk of tumor recurrence and an improved prognosis. However, there are numerous cases of WHO°1 MGMs showing clinically aggressive phenotypes with early and multiple recurrences while, in contrast, there are WHO°2 MGMs that do not recur [7,10]. Traditionally, the WHO classification for meningioma diagnostics was exclusively based on histopathological features. Due to intensive research, molecular markers have become increasingly relevant for the characterization and diagnosis of meningiomas, leading to further insights into the molecular pathogenesis of these tumors [7]. As a result, the latest update of the WHO classification of CNS tumors from 2021 has now incorporated several molecular factors for the clinical diagnosis of meningiomas. The most important molecular markers with clinicopathological significance that are based on tumor-subtype-specific genetic alterations are listed below (see Table 1) [4,11,12,13,14].

## 3. Genomic Landscape of Meningiomas

Tumor development is driven in part by copy number alterations (CNAs), which are somatic changes in chromosome structure referring to a gain or loss of copies of DNA segments. In numerous tumors, chromosomal aberrations have been described as a driving force for malignancy through the dysregulation of oncogenes and tumor suppressor genes, which is also true for meningiomas [15,16]. With the advances in next-generation sequencing (NGS) technologies, several common cytogenic and mutational alterations have been identified as molecular drivers of MGM pathogenesis. Prominent chromosomal aberrations in meningiomas are loss of chromosomes 22q, 1p and 14q, all of which are specifically associated with increased clinical aggressiveness. Other recurring chromosomal changes found in higher-grade tumors include the loss of chromosomes 4p, 6q, 7p, 9p, 10q, 11p, 14q and 18q. In addition, chromosomal gains of 17q and 20q were also frequently described in higher-grade meningiomas. In summary, WHO°2 and °3 tumors exhibit higher rates of genomic disruption, whereas WHO°1 MGMs often present the loss of chromosome 22q as the only recurring chromosomal aberration [15].

Compared to other cancer entities, meningiomas display a relatively low tumor mutational burden (TMB). Nevertheless, several genetic driver mutations have been discovered in meningiomas, of which the most prominent one is described for the tumor suppressor gene Neurofibromin-2 *(NF2)*. *NF2*, when mutated, accounts for the inherited genetic disorder Neurofibromatosis type 2, which leads to the development of schwannomas and meningiomas in patients [15,16]. The *NF2* gene, located on chromosome 22, encodes for the protein merlin, which plays a vital role in several cell proliferation and survival pathways. Inactivating mutations of the *NF2* gene are found in approximately 50% of meningiomas across all WHO grades. *NF2*-mutated tumors are more frequently located in the convexity, tend to be larger in size and display increased mitotic indices, and are therefore associated with increased malignancy [14,15].

The tumor-suppressor genes *CDKN2A* and *CDKN2B* are frequently mutated in numerous types of cancer, including meningiomas, and their products play important roles in cell cycle regulation [15]. In a large MGM cohort of 528 specimens, Sievers and colleagues found homozygous deletions of *CDKN2A/B* in 4.9% of all cases [17]. Importantly, homozygous loss of *CDKN2A/B* was only found in higher-grade tumors (27% in WHO°2 MGMs and 73% in WHO°3 MGMs) and turned out to be an independent prognostic factor for poor patient outcome. Hence, homozygous loss of *CDKN2A/B* is now listed as molecular factor for the diagnosis of WHO°3 MGMs in the latest WHO classification (see Table 1) [15,17].

Telomerase reverse transcriptase (*TERT*) promoter mutations present another useful biomarker in meningioma diagnostics [7,12]. Cell immortalization is one of the hallmarks of cancer that is caused by telomere maintenance through reactivation of the enzyme telomerase [18]. In malignant cells, activating mutations in the *TERT* promoter (*TERT*p) lead to the upregulation of *TERT* gene expression, and ultimately, increased telomerase activity [7,15,18]. *TERT*p mutations are found in meningiomas across all WHO grades but show substantial enrichment in aggressive subtypes (frequency of *TERT*p mutations: 4.7% in WHO°1, 7.9% in WHO°2 and 15.4% WHO°3), with a significantly reduced progression-free survival (PFS) in affected patients (14 months vs. 101 months, respectively). For improved classification, *TERT*p mutations have been incorporated, as well as molecular factors, for the diagnosis of WHO°3 meningiomas in the latest WHO classification from 2021 (see Table 1) [7,12].

Further frequent non-*NF2* driver mutations in meningiomas include oncogenic mutations in *TRAF7*, *KLF4*, *AKT1* and *SMO*, which are all mutually exclusive to *NF2* mutations. *TRAF7* mutations have been described in approximately 20–25% of all meningiomas and often co-occur with mutations in *KLF4* or *AKT1*, which are in turn both mutually exclusive to each other. Mutations in the gene Smoothened (*SMO*), which is involved in the hedgehog signaling pathway, are described in 3–5% of all meningiomas [15]. In 2013, Clark and colleagues first described an association between WHO grade, histological subtype and the anatomical location of meningiomas with distinct molecular features, including mutations in *TRAF7*, *KLF4*, *AKT1*, *SMO* and *NF2* [15,19]. Interestingly, meningiomas with mutations in *TRAF7*, *KLF4*, *AKT1* and *SMO,* as well as *POLR2A*, were later described by Nassiri and colleagues to have a more favorable patient prognosis compared to other genetic driver mutations [20]. Other mutations that are frequently described in meningiomas, but occur much rarer, are found in the genes *PIK3CA*, *PTEN*, *SMARCB1*, *SMARCE1*, *BAP1* and *FOXM1* but are not further discussed here [15].

## 4. Epigenetic Landscape of Meningiomas

In tumor biology, epigenetic reprogramming has become increasingly important and was even named by Douglas Hanahan in 2022 as a distinctive feature that enables the acquisition of characteristic abilities, which essentially lead to cancer development [21]. As part of epigenetic reprogramming, DNA methyltransferases catalyze the addition of methyl groups to cytosine-guanosine dinucleotides (CpGs), often enriched in so-called islands in gene promoters or other regulatory regions of the DNA, which ultimately influences gene transcription [22]. In tumor diagnostics, the analysis of non-mutational, tumor-specific, genome-wide DNA methylation patterns is a useful tool for the unambiguous identification of malignancies and their further stratification into clinically relevant molecular subtypes. In brain tumor diagnostics, DNA methylation-based classification is widely used and can be more precise compared to traditional histopathological analyses [14].

Several large studies have investigated genome-wide DNA methylation profiles in meningiomas, which overall have demonstrated improved stratification of tumors harboring a high recurrence risk while allowing for improved prediction of patient outcomes [20,22,23,24,25,26,27,28]. In 2017, Sahm and colleagues were the first to publish a DNA methylation-based classification system encompassing six defined methylation groups, which provided improved prediction of tumor biology and clinical outcomes [21]. The six determined methylation classes (MCs) benign-1/2/3 (ben-1/2/3), intermediate-A/B (int-A/B), and malignant (mal) presented each distinct features regarding genetic mutations, cytogenetic changes, histological subtypes and PFS of patients, and exceeded by far the WHO classification from 2016 for tumor outcome prediction [22,24]. Further developed from the work by Sahm et al. (2017) [24], Maas and colleagues published an integrated molecular-morphologic classification system where WHO grading, DNA methylation subgroups (here referred to as methylation families) and copy number variations (CNVs) were analyzed together in a total cohort of 3031 meningiomas, which showed significantly improved precision in meningioma stratification [27]. Altogether, these studies highlighted the relevance of DNA methylation profiles for meningioma biology and behavior and their usage as improved diagnostic tools, largely outperforming traditional histopathologic analyses. However, there is still an unmet need for a standardized implementation of DNA methylation analyses for meningioma diagnostics in the clinic, which could support tumor management and improve overall patient outcome [22].

## 5. Immunological Landscape of Meningiomas

Compared to other common CNS malignancies, meningiomas are not limited by the blood–brain barrier and are therefore more easily accessible to immune cells from the periphery [29]. The tremendous clinical success of immune checkpoint blockade (ICB) therapies in several other cancer entities has resulted in a great surge of interest on the tumor’s immunobiology, recognizing the importance of the immune system on cancer development and patient outcome [30]. In comparison to other tumor types, meningiomas are characterized by a low mutational burden and are regarded as lymphocyte-excluded tumors with a predominantly immunosuppressive microenvironment [30,31,32]. So far, several studies have investigated the immunological repertoire in meningiomas and found a complex immune cell infiltrate consisting of tumor-associated macrophages (TAMs), T cells, myeloid-derived suppressor cells, mast cells, natural killer (NK) cells and few B cells. In these analyses, the MGM immune microenvironment has been studied with traditional immunological methods like flow cytometry, immunohistochemistry (IHC) and multicolor immunofluorescence (IF) [33,34,35,36,37,38,39,40,41,42,43,44]. However, a limiting factor for most of these studies is the relatively small patient cohorts, consisting of merely 50 meningioma samples without a balanced WHO grade distribution, which overall represents a major drawback for their biological significance. Fortunately, some of these studies are characterized by large and clinically well-annotated patient cohorts, of which some are discussed in more detail below. Three recent studies also applied state-of-the-art methodologies, such as single-cell RNA-sequencing, which enabled a more in-depth characterization of the immune infiltrate in meningiomas, although with limited informative value due to their small sample size [20,26,45]. Further, a number of clinical trials are currently investigating the efficacy of ICB therapies in meningiomas, promoting a role for immunotherapeutic strategies for the clinical management of this tumor entity [29,31].

## 6. PD-L1 Expression in Meningiomas

The immune checkpoint molecule programmed death-ligand 1 (PD-L1) is known to be a prognostic marker in various cancer entities and has also been investigated in meningiomas [30]. In 2014, Du and colleagues analyzed lymphocyte infiltration and the expression of PD-L1 (CD274) in a large cohort of 291 meningioma samples (n = 195 WHO°1, n = 73 WHO°2 and n = 23 WHO°3) [40]. Analysis of PD-L1 using immunohistochemistry in tissue microarrays (IHC/TMA) and RNAscope, respectively, revealed significantly increased PD-L1 levels in WHO°2 and °3 meningiomas, both for mRNA and protein expression [40]. In a subsequent study, Han and colleagues analyzed PD-L1 expression together with CD68 expression (a macrophage marker) in a cohort of 96 meningioma specimens (n = 16 WHO°1, n = 62 WHO°2 and n = 18 WHO°3) using IHC/TMA [42]. Interestingly, the authors reported higher PD-L1 expression in CD68-negative cells (likely tumor cells) in WHO°2 and °3 tumors and described further high PD-L1+/CD68- staining as an independent prognostic marker for poor overall survival in meningioma patients [30,42]. In 2020, a study conducted by Karimi and colleagues investigated PD-L1 expression in a cohort of 93 meningiomas (n = 41 WHO°1, n = 43 WHO°2 and n = 9 WHO°3) using IHC in whole-tissue sections and reported PD-L1 positivity in 43% of all cases, with a significantly increased PD-L1 expression in higher-grade tumors. Moreover, in both univariate and multivariate analyses, PD-L1 protein expression was found to be an independent prognostic marker for poor PFS in meningioma patients [41].

## 7. Tumor-Infiltrating T Lymphocytes in Meningiomas

Several smaller studies reported overall low numbers of tumor-infiltrating lymphocytes in meningiomas [34,38]. Using IHC/TMA, Du and colleagues reported meningioma lymphocyte infiltrates to be predominantly composed of T cells as opposed to B cells [30,40]. The authors found a significant decrease for T cell infiltration in WHO°3 meningiomas compared to WHO°1 tumors for both CD4+ T helper cell and CD8+ cytotoxic T cell subsets. Further, FOXP3+ cells (referring to regulatory T cells) were reported to be significantly increased in higher-grade meningiomas. In a study from our group, we analyzed T cell infiltration using multicolor immunofluorescence in whole-tissue sections and semi-automated quantitative analysis in a large cohort of 202 clinically well-annotated meningiomas [33]. Analysis revealed an extremely heterogeneous T cell infiltration across tumors with a median T cell infiltration of 0.59% per total cell count (TCC) for primary meningiomas (pMGMs n = 123; n = 33 WHO°1, n = 64 WHO°2 and n = 26 WHO°3) and of 0.33% per TCC for recurrent meningiomas (rMGMs n = 79; n = 10 WHO°1, n = 33 WHO°2 and n = 36 WHO°3). For primary tumors, we found no significant differences among WHO grades for total CD3+ T cells (CD3+) and T helper cells (CD3+CD8-FOXP3-), as well as cytotoxic T cells (CD3+CD8+FOXP3-). Nevertheless, in the survival analysis, a higher infiltration of cytotoxic T cells was associated with an improved PFS in meningioma patients and, moreover, turned out to be an independent prognostic factor in a multivariate analysis. When compared to primary meningiomas, recurrent tumors showed lower T cell infiltrates, including total CD3+ T cells, T helper cells and cytotoxic T cells, with the latter subtype being significantly decreased in higher-grade tumors. In line with previous findings, we also found regulatory T cells (CD3+CD8-FOXP3+) to be enriched in WHO°2 and °3 tumors, both for primary and recurrent meningiomas. In addition, co-staining for the immune checkpoint molecule PD1 revealed a significantly reduced proportion of CD3+CD8+PD1+ T cells within the CD3+ T cell population (% of CD3+) in higher-grade tumors, which was true both for primary and recurrent meningiomas. Further, in a subsequent multivariate analysis we found higher proportions of CD3+PD1+ T cells (% of CD3+) as an independent predictor of pro-longed PFS in meningioma patients. Importantly, despite their overall low T cell infiltration rate, our analysis identified higher infiltration of cytotoxic T cells (CD3+CD8+FOXP3-) and higher proportions of CD3+PD1+ T cells as novel independent prognostic factors. Together, these findings encourage an improved selection of meningioma patients who might benefit from ICB therapies in the future [33].

## 8. Tumor-Associated Macrophages in Meningiomas

TAMs have been reported to represent the main immune cell population in various brain malignancies [46,47]. In meningiomas, several studies investigated the myeloid cell compartment and reported the presence of TAMs [34,35,36,37,42,43,44,45,48,49]. In a small study exerted by Proctor and colleagues, TAM infiltration was analyzed in a cohort of 30 meningioma samples (n = 16 WHO°1, n = 12 WHO°2 and n = 2 WHO°3) using multicolor IF staining for CD68+ and CD163+ (M2-like macrophage marker). There, TAMs accounted for approximately 18% of total cells in tumor tissue and more than 80% of infiltrating TAMs presented a pro-tumoral M2-like phenotype (CD68+CD163+ cells) [35]. In 2021, Yeung and colleagues examined the immune cell compartment in silico using CIBERSORTx, applying gene signatures of 22 immune cell subsets to a dataset of 201 cases of meningiomas with whole-exome sequencing and gene expression analyses. Overall, RNA deconvolution revealed a myeloid-enriched immune compartment with high proportions of monocytes and M2-like macrophages across meningioma samples but, unfortunately, without further distinction on clinical parameters [49]. In another study, Yeung and colleagues analyzed the TAM compartment in a cohort of 73 meningioma specimens (n = 56 WHO°1, n = 13 WHO°2 and n = 4 WHO°3) by multicolor IF using TMAs. The analysis revealed a heterogeneous TAM infiltration across samples, with more than 50% of TAMs displaying a M2-like phenotype (CD68+CD163+ cells). Interestingly, the authors observed no significant differences in TAM and M2-like TAM infiltration rates when contrasting benign (WHO°1) and atypical/anaplastic (WHO°2/3) meningiomas [43], which is not in line with the previous findings by Proctor et al. but could be due to differences in study design and overall limited numbers of higher-grade tumors in both analyses [35]. However, altogether, these studies on the myeloid cell compartment described a large infiltrate of TAMs and immunosuppressive pro-tumoral M2-like TAMs in meningiomas, suggesting a potential role for these immune cells in tumor development and disease progression. Nevertheless, further analyses in larger and well-balanced tumor cohorts are needed to allow a more in-depth characterization of TAM infiltration, polarization and their functional role in meningiomas, especially with regard to tumor behavior and patient outcome.

## 9. Immunotherapy for Meningiomas

Due to the clinical success of T cell-based ICB therapies in several other cancer types, the question has long been raised whether immunotherapy could also represent a potential therapeutic option for meningiomas, especially for tumors refractory to standard treatment modalities [29,31]. Due to enormous research advances, a plethora of immunotherapeutic approaches have been approved for clinical practice in various tumor types, and many more are still in pre-clinical and clinical development [31]. In meningiomas, most clinical trials have focused so far on immune checkpoint inhibition, targeting the checkpoint molecules PD1/PD-L1 or CTLA4, either as monotherapy or in combination with radiotherapy. Several clinical trials on ICB therapy, enrolling patients with clinically aggressive meningiomas, are still ongoing and are summarized below (see Table 2) [29,31]. To induce a favorable clinical response to immune checkpoint inhibition in cancer patients, several prerequisites need to be met: (1) a higher tumor mutational burden with the presence of tumor-specific neo-antigens, (2) a higher intra-tumoral infiltration of T lymphocytes and (3) expression of ICB targets [50]. As summarized in the previous sections, meningiomas largely do not fulfill these essential criteria for eliciting clinically meaningful responses through ICB therapy, as they are generally characterized by a low tumor mutational burden, lower numbers of tumor-infiltrating T cells and heterogeneous expression of immune checkpoint molecules [29,30,31]. Another limitation of the presented clinical trials is the exclusive enrollment of patients with clinically aggressive meningiomas whose tumors have been heavily pretreated and therefore likely have a highly immunosuppressive microenvironment. Given these considerations, it is no surprise that results from clinical trials for T cell-based ICB therapies have been, so far, largely disappointing for meningioma patients [33]. Therefore, future clinical trials of immunotherapy should likely consider an improved stratification for enrolling meningioma patients based on the individual’s tumor microenvironment.

Further, a major challenge in meningioma research is the limited number of mouse models that are available, especially for the investigation of immunotherapeutic approaches. Frequently used preclinical models of meningioma are heterotopic and orthotopic xenograft models. Intracranial MGM xenograft models implanting tumor cells from established MGM cell lines are the current gold standard for assessing treatment efficacy in vivo. However, importantly, these models rely on immunocompromised mice and lack, therefore, a physiological TME, which may ultimately compromise drug efficacy. To overcome this limitation, a few genetically engineered mouse models (GEMMs) of meningioma have been successfully established but are not widely used, as they are expensive, time-consuming and complex [51]. Novel syngeneic orthotopic allograft models using immunocompetent wildtype mice may present the closest models of sporadic meningiomas, in particular for examining immunotherapies [52]. In 2021, Yeung and colleagues were the first to examine a macrophage-targeting approach in an immune-competent syngeneic mouse model of meningioma using anti-CSF1/CSF1R immunotherapy [49], which has been shown to induce anti-tumoral responses in other brain malignancies, such as glioma [53,54,55]. In their study, the authors reported that treatment of murine meningiomas using anti-CSF1/CSF1R monoclonal antibodies, in contrast to anti-PD1 therapy, was efficacious and inhibited tumor growth [49]. Considering the high numbers and immunosuppressive polarization of TAMs in human meningiomas, targeting macrophages might present an alternative promising immunotherapeutic treatment strategy for patients in the near future [31,35,43,49]. Fortunately, in recent years, a plethora of TAM-targeting drugs have entered pre-clinical and clinical testing for cancer immunotherapy, which will hopefully soon be investigated in suitable mouse and ex vivo models of meningioma as well.

**Table 2:** Overview on clinical trials with ICB therapy in patients with clinically aggressive meningiomas [31,56].

Clinical Trial Identifier	Design	Treatment	Checkpoint Molecule	N	Status
NCT02648997	Two arms, sequential assignment phase 2	1. Nivolumab 2. Radiotherapy followed by nivolumab with ipilimumab	1. PD1 2. PD1/CTLA4	50	Recruiting
NCT03016091	Single arm phase 2	Pembrolizumab	PD1	25	Unknown
NCT03173950	Basket trial phase 2	Nivolumab	PD1	180 ^a^	Recruiting
NCT03267836	Single arm phase 1b	Proton radiation therapy with avelumab	PD-L1	9	Terminated
NCT03279692	Single arm phase 2	Pembrolizumab	PD1	26	Active
NCT03604978	Randomized open label phase 1/2	Nivolumab and multi-fraction stereotactic radiosurgery with or without ipilimumab	PD1/CTLA4	38	Recruiting
NCT04659811	Single arm phase 2	Pembrolizumab and stereotactic radiosurgery	PD1	37	Recruiting

^a^ includes other cancer entities.

## 10. Immunogenetics and Immunoepigenetics of Meningiomas

To date, few attempts have been made to decipher the complex associations between the molecular pathogenesis and the immune microenvironment of meningiomas. In 2013, Domingues and colleagues examined the cytogenetic and gene expression profile as well as immune infiltration in a cohort of 75 meningioma specimens [44]. Interestingly, the authors described a clinically relevant subgroup of meningiomas with better patient outcome harboring isolated monosomy 22/del (22q), which was significantly associated with higher TAM and NK cell numbers [30,44]. In a study from 2020, Zador and colleagues applied a systems biology approach to analyze the immune microenvironment in skull base and convexity meningiomas (n = 107 MGMs) [57]. There, network analysis of bulk tumor RNA-sequencing data revealed unique cytokine-cell networks between the two analyzed subgroups: Skull base MGMs were highly infiltrated by monocytes, while convexity MGMs were dominated by mast cells and neutrophils, suggesting a link between the anatomical location of meningiomas and the tumor’s immune microenvironment [30,57].

In recent years, two large-scale studies from independent research groups have identified different molecular groups of meningiomas by integrating molecular data from various state-of-the-art methodologies [20,26]. In each study, the molecular groups were characterized by distinct molecular features, including *NF2* status, mutational status, copy number alterations, methylation profile and transcriptional programs, as well as clinical outcome (PFS) [58]. Interestingly, despite the differences in nomenclature, the authors independently identified, in both studies, a immunological molecular group with more favorable clinical outcomes and high biological similarities [20,26,58]. In the study from 2021, Nassiri and colleagues included 121 meningioma specimens in their multi-omics analyses and utilized a multilayered clustering approach to uncover four molecular groups of meningioma. One molecular group was designated as “immunogenic”, in which meningiomas were characterized by *NF2* mutations and the loss of chromosome 22q as well as enrichment in transcriptomic pathways involved in immune signaling and regulation [20]. In another study from 2022, comprising 565 meningioma specimens, Choudhury and colleagues identified a similar molecular group designated as “immune-enriched”, which was characterized by the presence of lymphatic vessels, increased immune infiltration and HLA expression, as well as a loss of chromosome 22q (*NF2*) and a gain of chromosome 6p (*HLA*) as genetic features [26]. In both studies, the molecular meningioma classification by far exceeded the standard WHO classification in terms of biological relevance and clinical features [20,26,58]. Importantly, the molecular classification by Nassiri and colleagues was superior in predicting time to recurrence compared to WHO grading and the previously described DNA methylation-based classification by Sahm and colleagues from 2017, suggesting its use to improve the timing of an adjuvant treatment [20,24]. In summary, these studies have first illustrated a complex interplay between the molecular pathogenesis and the immune microenvironment in meningiomas, highlighting the need for an integrated molecular classification of meningiomas that also considers the importance of the tumor’s immunobiology [27].

## 11. Conclusions and Future Perspectives

Traditionally, meningiomas have been viewed as benign and slow-growing tumors, but there has always been a substantial subset of clinically aggressive phenotypes whose clinical management remains challenging to date. These tumors show early and multiple recurrences, range from WHO°1 to WHO°3 and are refractory to current standard treatment modalities. With a plethora of research advances, several molecular markers have been identified that play essential roles in MGM tumorigenesis, some of which are now even recognized in the latest update of the WHO classification of CNS tumors from 2021 [11,12]. Moreover, several independent research groups have contributed to an improved molecular classification of meningiomas by identifying clinically relevant molecular subgroups according to genetic and epigenetic profiles, transcriptional programs, protein expression and patient outcome [20,24,25,26,27,28,58]. Altogether, these studies provided relatively similar findings on genome-wide DNA methylation profiles and molecular subgroups of meningiomas but with the major drawback of a lack of standardization and thus a lack of implementation in the clinical setting, which is, however, highly required to improve the clinical management of these tumors. To date, the use of molecular markers is still solely advocated by the WHO classification in uncertain cases to refine MGM diagnosis but is not a mandatory requirement [12]. Although great progress has been made in deciphering the molecular pathogenesis of meningiomas, the heterogeneity of these tumors still hinders the successful development and implementation of targeted therapies in the clinic, and there are still no systemic treatment options available for patients with challenging tumors [6]. With the breakthrough of T cell-based cancer immunotherapies, research efforts in the field of immune profiling of meningiomas have steadily increased, but knowledge about the functional impact of the immune microenvironment remains limited. Several studies have described a pre-dominantly immunosuppressive microenvironment in meningioma, particularly in higher-grade tumors, characterized by high numbers of immunosuppressive macrophages, low numbers of tumor-infiltrating T lymphocytes and higher expression of the immune checkpoint molecule PD-L1 [30,31,32,33,35,40,41,43,49]. Some of these immunological features, such as overall PD-L1 expression in MGM tissue as well as infiltration of cytotoxic (CD3+CD8+FOXP3-) and PD-1 expressing (CD3+PD-1+) T cells, were individually reported to be independent prognostic factors for predicting patient outcomes [33,41]. In prospective clinical trials, these findings might facilitate the selection of patients, who may benefit from immunotherapeutic approaches. However, additional steps are required before these immunological features can be readily used as biomarkers in the clinical practice, which include, first, their biological validation in novel cohorts and, second, the establishment of a standardized and easy-applicable method for routine tumor diagnostics [58]. Importantly, compared to other brain malignancies, research into the immunobiology of meningiomas is still in its relative infancy [30]. Elucidating a clear association between the molecular profile of these tumors and their immune microenvironment remains the subject of current and future investigations. Tying together these loose ends in meningioma research could hopefully lead to novel therapeutic breakthroughs and ultimately the improved treatment of patients with clinically aggressive meningiomas in the near future.

## Figures and Tables

**Table 1:** Meningioma classification: WHO grades, histological subtypes and molecular markers. WHO CNS classification from 2021 [4,11,12,13,14].

WHO Grade	Histological Subtype	Molecular Markers
1	Meningothelial Fibrous Transitional Psammomatous Angiomatous (vascular) Microcystic Secretory Lymphoplasmacyte-rich Metaplastic	Mutation: *TRAF7*, *KLF4*, *AKT1*, *SMO*, *PIK3CA* Mutation: *NF2* Mutation: *NF2*, *TRAF7*, *KLF4*, *AKT1*, *SMO*, *PIK3CA* Mutation: *TRAF7*, *KLF4*, *AKT1*, *SMO*
2	Clear cell Chordoid Atypical	Mutation: *NF2*, *SMARCE1* Mutation: *NF2* Mutation: *NF2*
3	Rhabdoid Papillary Anaplastic	Mutation: *BAP1* Mutation: *NF2*, *BAP1* Mutation: *NF2*, *TERT*p; Chrom alt: *CDKN2A/B* loss

*TERT*p = *TERT* promoter, chrom alt = chromosomal alteration.

## Abbreviations

Ben	Benign
CNA	Copy number alteration
CNS	Central nervous system
CNV	Copy number variation
CpG	Cytosine-guanosine dinucleotide
GEMMs	Genetically engineered mouse models
ICB	Immune checkpoint blockade
IF	Immunofluorescence
IHC	Immunohistochemistry
Int	Intermediate
Mal	Malignant
MC	Methylation class
MGM	Meningioma
*NF2*	Neurofibromin-2
NGS	Next-generation sequencing
NK cell	Natural killer cell
PD-L1	Programmed death-ligand 1
PFS	Progression-free survival
pMGM	Primary meningioma
rMGM	Recurrent meningioma
*SMO*	Smoothened
TAM	Tumor-associated macrophage
*TERT*	Telomerase reverse transcriptase
*TERT*p	*TERT* promoter
TMA	Tissue microarray
TMB	Tumor mutational burden
WHO	World Health Organization
